# Delayed diagnosis of pneumonia in the emergency department: factors associated and prognosis

**DOI:** 10.3389/fmed.2023.1042704

**Published:** 2023-05-12

**Authors:** Maria Bouam, Christine Binquet, Florian Moretto, Thibault Sixt, Michèle Vourc’h, Lionel Piroth, Patrick Ray, Mathieu Blot

**Affiliations:** ^1^Emergency Department, Dijon-Bourgogne University Hospital, Dijon, France; ^2^CHU Dijon-Bourgogne, INSERM, Université de Bourgogne, CIC 1432, Module Épidémiologie Clinique, Dijon, France; ^3^LabEx LipSTIC, University of Burgundy, Dijon, France; ^4^Department of Infectious Diseases, Dijon-Bourgogne University Hospital, Dijon, France; ^5^Biostatistics and Bioinformatics Department (DIM), Dijon-Bourgogne University Hospital, Dijon, France; ^6^Lipness Team, INSERM Research Centre LNC-UMR1231 and LabEx LipSTIC, University of Burgundy, Dijon, France

**Keywords:** community-acquired pneumonia, delayed diagnosis, antibiotic therapy, outcomes, mortality, pneumonia, emergency department

## Abstract

**Introduction:**

Whether a delayed diagnosis of community-acquired pneumonia (CAP) in the emergency department (ED) is associated with worse outcome is uncertain. We sought factors associated with a delayed diagnosis of CAP in the ED and those associated with in-hospital mortality.

**Methods:**

Retrospective study including all inpatients admitted to an ED (Dijon University Hospital, France) from 1 January to 31 December 2019, and hospitalized with a diagnosis of CAP. Patients diagnosed with CAP in the ED (*n* = 361, early diagnosis) were compared with those diagnosed later, in the hospital ward, after the ED visit (*n* = 74, delayed diagnosis). Demographic, clinical, biological and radiological data were collected upon admission to the ED, as well as administered therapies and outcomes including in-hospital mortality.

**Results:**

435 inpatients were included: 361 (83%) with an early and 74 (17%) with a delayed diagnosis. The latter less frequently required oxygen (54 vs. 77%; *p* < 0.001) and were less likely to have a quick-SOFA score ≥ 2 (20 vs. 32%; *p* = 0.056). Absence of chronic neurocognitive disorders, of dyspnea, and of radiological signs of pneumonia were independently associated with a delayed diagnosis. Patients with a delayed diagnosis less frequently received antibiotics in the ED (34 vs. 75%; *p* < 0.001). However, a delayed diagnosis was not associated with in-hospital mortality after adjusting on initial severity.

**Conclusion:**

Delayed diagnosis of pneumonia was associated with a less severe clinical presentation, lack of obvious signs of pneumonia on chest X-ray, and delayed antibiotics initiation, but was not associated with worse outcome.

## Introduction

Community-acquired pneumonia (CAP) remains a leading cause of death worldwide. Patients with CAP are frequently managed in the emergency department (ED), representing for instance 85% of hospitalized patients with pneumococcal pneumonia (1). It has been shown in experimental models of pneumonia, as well as in patients admitted for CAP, that delaying antibiotic administration may be associated with worse outcomes (2–4). Prompt diagnosis after hospital admission is essential for the quick initiation of antimicrobial therapies, and for avoiding underdiagnosis and premature discharge. Previous studies have reported that discordances between admission and discharge diagnoses are frequent and associated with worse outcomes (5, 6). Among patients with an infection, a misdiagnosed infection site is associated with a 10% increase in in-hospital mortality (7).

Pneumonia is defined as the association of clinical (infectious and respiratory) and radiological (new infiltrate) signs (8–10). However, the diagnosis is not always obvious due to the heterogeneity of clinical presentations related to various pathogens (e.g., virus, bacteria, and co-infections) (9) and patient characteristics (e.g., mild symptoms and afebrile pneumonia in the elderly or immunocompromised patients) (11), or because of the absence of an infiltrate on chest radiography (12). Some studies have reported that 16 to 35% of patients admitted to the ED with a CAP diagnosis had an alternative diagnosis upon discharge (13–17). However, to the best of our knowledge, no study has investigated the factors associated with misdiagnosis of pneumonia upon admission to the ED in patients with a diagnosis of CAP on discharge. This situation is relevant since a misdiagnosis of CAP upon ED admission could be associated with a delayed initiation of appropriate therapy and inadequate referrals to other hospital wards. However, whether the delay of antibiotic administration could impact outcomes during CAP is challenged by a recent study showing that a strict 4 h threshold for antibiotic administration in all patients had no significant effect on the outcomes (18).

This study aimed to identify, among inpatients with a discharge diagnosis of CAP, the prevalence and factors associated with a non-pneumonia diagnosis in the ED and whether this delay in diagnosis was associated with patient outcome.

## Methods

### Study design

We conducted an observational retrospective study including all patients admitted to the ED of the 1,200-bed Dijon University Hospital (France) from 1 January 2019 to 31 December 2019, and hospitalized for at least 48 h with a discharge diagnosis of CAP. Inclusion criteria were as follows: age >18 years, admission to ED and subsequent hospitalization for at least 48 h with a discharge diagnosis of CAP.

Hospital stays for pneumonia were first identified by primary diagnoses with the International Classification of Diseases (ICD)-10 codes A481, J13, J14, J100, J110, J120, J121, J122, J123, J128, J129, J150, J151, J152, J153, J154, J155, J156, J158, J159, J168, J170, J180, J181, J188, J189, J440, J690, J851 in the French hospital discharge database (PMSI, *Programme de médicalisation des systèmes d’information*). Then, the accuracy of the diagnosis was checked in individual medical files by a trained clinician. Patients were eligible if they had a diagnosis of community-acquired pneumonia (CAP) made by the attending clinician, based on clinical signs of pneumonia, a radiological pulmonary infiltrate and diagnosed within 48 h following admission at the hospital. If not confirmed, or if the diagnosis was made after 48 h of hospitalization (nosocomial pneumonia), patients were excluded from the analysis. Then, patients with a diagnosis of CAP from the ED (early diagnosis) were compared with those whose diagnosis of CAP was made in the hospital ward, after the ED visit (delayed diagnosis).

### Patient consent

Study protocol and data collection were registered with the French national data protection authority (*Commission nationale de l’informatique et des liberté*s) and are in accordance with French (Loi Informatique et Liberté n°78-17 du 6 janvier 1978) and European (GRPD EU 2016/679) regulations on data protection and patient information (Commitment of compliance MR004 n°2,210,228 of 3 December 2018). Informed consent was waived given the non-interventional study design.

### Data collected

The following data were collected upon admission to the ED: the main reasons for presenting to the ED, demographic, clinical and biological variables, and radiological examinations and findings. Quick-Sepsis-related Organ Failure Assessment (SOFA) scores were calculated when the included variables were reported. Administered therapies (oxygen, antibiotics, vasopressors, non-invasive or invasive ventilation), and outcomes (transfer to intensive care unit, number of days without antibiotics, or hospitalization during the first 30 days after admission, in-hospital mortality) were collected. Required oxygen flow rate in the ED was scored as follow: (1) no oxygen; (2) oxygen administration ≤3 L/min; (3) 3–10 L/min; and (4) >10 L/min or non-invasive or invasive ventilation.

### Statistical analyses

Continuous variables were expressed as means ± standard deviation (SD) or medians and inter-quartile ranges (IQR) depending on their distribution, and categorical variables as frequencies and percentages. The Student’s *t*-test was used to compare means for quantitative variables with Gaussian distribution, and the Wilcoxon Mann–Whitney test was used to compare medians in case of non-Gaussian distribution or heterogeneity of variances. The normality of variable distribution was checked with the Shapiro-Wilks test. To account for potential confounders, multivariable logistic regression was performed to identify factors independently associated with a delayed diagnosis of CAP after ED care. All variables associated with a delayed diagnosis with a value of *p* < 0.2 in bivariate analyses and with less than 10% of missing data were accounted for. First, two models were built, the first one considering chest X-ray-related variables (examination performed, and findings; model 1), the second one without this information (model 2). Finally, another model was built to test if a delayed diagnosis was independently associated with in-hospital mortality. Correlations between variables were checked before their inclusion in the model in order to avoid collinearity. Log-linearity was tested for each continuous variable using fractional polynomials (19). A backward step-by-step selection process was then applied to obtain the final model. Interactions between selected variables were systematically tested. Results were expressed as odds ratios (OR) and 95% confidence intervals (CI). A value of *p* <0.05 was considered statistically significant. Analyses were performed using Stata v15.1 (StataCorp LLC, College Station, TX, USA) and GraphPad Prism software version 9.1.1 (San Diego, CA, USA).

## Results

**Figure 1:**
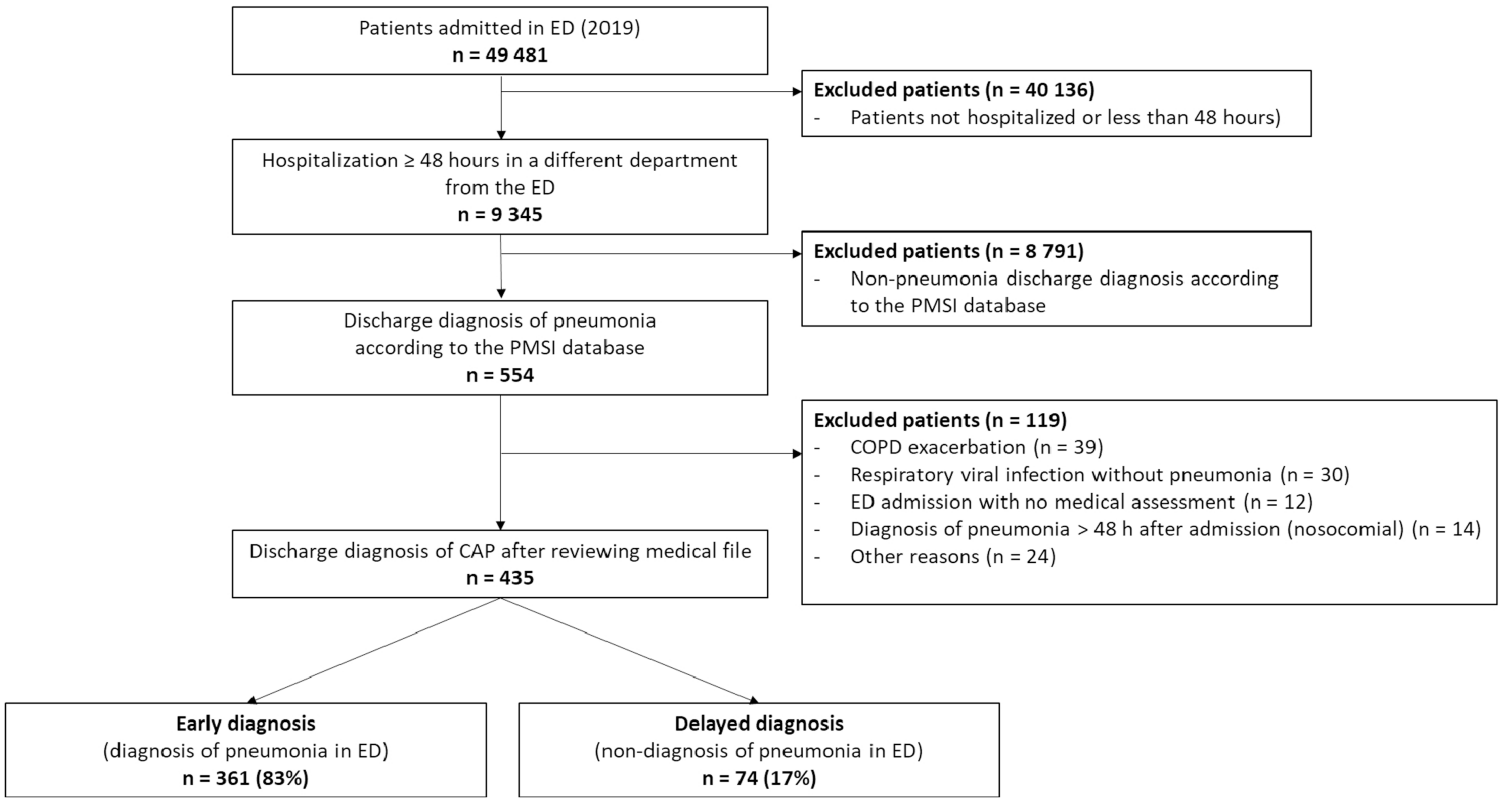
Flowchart NB: CAP, community-acquired pneumonia; ED, emergency department; PMSI, *programme de médicalisation des systèmes d’information.*

Among the 49,481 patients admitted to the ED of the Dijon University Hospital in 2019, 9,435 were hospitalized ≥48 h in a hospital ward, 554 had a discharge diagnosis of pneumonia according to the PMSI database, and, after a review of the medical files, 435 were discharged with a final diagnosis of CAP according to the clinician (Figure 1). The characteristics of the 435 patients are reported in Table 1. Overall, patients were aged 76 (±16) years, 39% were female, and they presented a median of 2 comorbidities (interquartile range [IQR] = 1–3) per patient. All patients had chest imaging (chest X-ray or CT scan) during the first 48 h of hospitalization, and 418 of them (96%) within the ED. Among them, 361 (83%) were diagnosed with pneumonia upon ED admission (early diagnosis) and 74 (17%) had a delayed diagnosis (Figure 1). The main reasons for presenting to the ED are described in Supplementary Table 1. Patients with a delayed diagnosis were more frequently referred to the ED after a fall [30 (8%) vs. 12 (16%); *p* = 0.036] compared to patients with an early diagnosis. For patients with a delayed diagnosis, the main alternative diagnoses retained in the ED were bronchitis/exacerbation of *chronic obstructive pulmonary disease* (COPD) or asthma (*n* = 13), viral respiratory infections with no pneumonia (*n* = 10) and an unexplained fever (*n* = 13; Supplementary Table 2). Patients with a delayed diagnosis of pneumonia stayed longer in the ED [median (IQR) 7.4 (5.53–10.5) vs. 6.5 (5.2–8.9) hours in patients with early diagnosis, *p* = 0.034].

**Table 1:** Characteristics of community-acquired pneumonia patients included according to the early or delayed diagnosis of pneumonia in the emergency department.

	All	Early	Delayed	*p*-value
	*n* = 435	*n* = 361	*n* = 74	
Demographics				
Age, mean (±SD)	76 (±16)	76 (±16)	76 (±13)	0.869
Female sex, *n* (%)	171 (39)	136 (38)	35 (47)	0.123
BMI (kg/m^2^), median (IQR) (*n* = 347)	25 (21-28)	25 (21-28)	25 (21-28)	0.831
Comorbidities				
Chronic lung disease, *n* (%)	159 (37)	130 (36)	29 (39)	0.605
Cardiovascular disease, *n* (%)	73 (17)	59 (16)	14 (19)	0.589
Chronic kidney disease, *n* (%)	51 (12)	40 (11)	11 (15)	0.357
Chronic liver disease, *n* (%)	54 (12)	38 (11)	16 (22)	0.008
Chronic neurological disorders, *n* (%) (*n* = 433)	192 (44)	169 (47)	23 (31)	0.013
Chronic neurocognitive disorders, *n* (%) (*n* = 433)	88 (20)	79 (22)	9 (12)	0.055
Diabetes mellitus, *n* (%)	93 (21)	76 (21)	17 (23)	0.714
Inflammatory/immune disorders, *n* (%) (*n* = 433)	88 (20)	79 (22)	9 (12)	0.055
Solid cancer, *n* (%)	22 (5)	18 (5.0)	4 (5)	0.881
Hematological cancer, *n* (%)	37 (9)	31 (9)	6 (8)	0.893
Immunodepression, *n* (%)	36 (8)	29 (8)	7 (10)	0.685
Number of comorbidities, median (IQR)	2 (1-3)	2 (1-3)	2 (1-3)	0.146
Tobacco use, *n* (%)	54 (12)	44 (12)	10 (14)	0.753
Emergency department care				
First medical contact between 6 pm and 8 am (“off hours”), *n* (%)	181 (42)	156 (43)	25 (34)	0.134
Elapsed time between admission and first medical contact (min), median (IQR)	51 (24–106)	51 (23–99)	52 (26–133)	0.236
Patient complaint				
Symptom duration before admission, median (IQR) (*n* = 424)				0.779
<48 h	218 (51)	183 (52)	35 (48)	
48 h to 7 days	107 (25)	88 (25)	19 (26)	
≥7 days	99 (23)	80 (23)	19 (26)	
Cough, *n* (%)	234 (54)	195 (54)	39 (53)	0.836
Dyspnea, *n* (%) (434/360/74)	280 (65)	244 (68)	36 (49)	0.002
Sputum, *n* (%)	130 (30)	106 (29)	24 (32)	0.599
Chest pain, *n* (%)	65 (15)	55 (15)	10 (14)	0.705
Upper respiratory signs, *n* (%)	15 (3)	13 (4)	2 (3)	0.700
Myalgia, *n* (%)	15 (3)	13 (4)	2 (3)	0.700
Physical exam at admission to ED				
Body temperature (°C, zenith), median (IQR) (*n* = 429)	38.1 (37.3–38.9)	38.2 (37.4–38.9)	37.7 (37.0–38.8)	0.055
Body temperature (zenith) ≥ 38°C, *n* (%)	233 (54)	201 (56)	32 (43)	0.051
Systolic blood pressure (mmHg, nadir), mean (±SD)	117 (24)	115 (23)	122 (30)	0.031
Heart rate (/min, zenith), median (IQR)	99 (83–114)	100 (83–115)	95 (82–112)	0.138
Glasgow score < 15, *n* (%)	100 (23)	87 (24)	13 (18)	0.224
Respiratory rate ≥ 22/min, *n* (%) (356/296/60)	197 (55)	168 (57)	29 (48)	0.231
Quick SOFA, median (IQR) (*n* = 356)	1 (0–2)	1 (0–2)	1 (0–1)	0.133
Quick SOFA ≥2, *n* (%) (*n* = 356)	108 (30)	96 (32)	12 (20.0)	0.056
Oxygen requirement				<0.001
No oxygen	117 (37)	83 (23)	34 (46)	
1–3 L/min	131 (30)	110 (30)	21 (28)	
4–10 L/min	119 (27)	104 (29)	15 (20)	
>10 L/min or (non)invasive ventilation	68 (16)	64 (18)	4 (5)	
Biological findings at admission to ED				
Hemoglobin (g/dl), mean (±SD)	12.6 (2.2)	12.7 (2.2)	12.1 (2.1)	0.046
Platelets (×10^9^/mm^3^), median (IQR)	229 (173–291)	229 (175–292)	229 (170–279)	0.905
Leucocytes (×10^3^/mm^3^), median (IQR)	12.0 (8.6–15.7)	12.1 (9.0–15.8)	11.1 (7.9–14.5)	0.211
Neutrophils (×10^3^/mm^3^), median (IQR)	9.5 (6.5–13.3)	9.7 (6.7–13.4)	8.2 (5.6–12.5)	0.086
Lymphocytes (×10^3^/mm^3^), median (IQR)	0.92 (0.55–1.36)	0.90 (0.55–1.36)	1.03 (0.56–1.54)	0.299
Creatininemia (μmol/L), median (IQR)	87.0 (67.0–121.0)	88.0 (67.0–120.0)	83.5 (66.0–123.5)	0.888
C-reactive protein (mg/L), median (IQR) (*n* = 420)	101.0 (40.1–189.0)	103.0 (40.9–208.0)	97.1 (30.9–143.0)	0.156
Procalcitonin (μg/L), median (IQR) (*n* = 218)	0.4 (0.2–1.8)	0.4 (0.2–1.8)	0.2 (0.1–2.0)	0.189
Radiological exams at admission to ED				
Chest X-ray performed, *n* (%)	395 (91)	333 (92)	62 (84)	0.022
Chest X-ray with signs of pneumonia according to the emergency physician’s medical report, *n* (%) (*n* = 352)	245 (70)	241 (81)	4 (8)	< 0.001
Pulmonary CT-scan performed, *n* (%)	57 (13)	56 (16)	1 (1)	0.001
Chest imagery performed, *n* (%)	418 (96)	355 (98)	63 (85)	< 0.001

NB: in case of unavailable data, the number of data considered in the analysis was reported (*n* = X).

### Comparison of patient characteristics according to the delay in pneumonia diagnosis

Compared to patients with an early diagnosis, those with a delayed diagnosis were significantly more likely to have chronic liver diseases and less likely to have neurocognitive chronic disorders (Table 1). Patients with a delayed diagnosis also had significantly less dyspnea, higher mean systolic blood pressure, marginally lower median body temperature, and lower mean hemoglobin levels than patients with an early diagnosis. Patients with a delayed diagnosis had a quick-SOFA score ≥ 2 less often (20 vs. 32%; *p* = 0.056). Furthermore, they were less likely to have had chest imagery (85 vs. 98%; *p* < 0.001), whether it was a chest X-ray (*p* = 0.022) or a CT-scan (*p* < 0.001). Among patients who had available interpretation of chest X-ray, radiological signs of pneumonia were significantly less frequently reported in those with a delayed diagnosis (8 vs. 81%; *p* < 0.001; Table 1).

### Factors independently associated with a delayed diagnosis of pneumonia in the ED

Multivariable logistic regression showed that factors independently associated with a delayed diagnosis of CAP in the ED were the absence of neurocognitive chronic disorders, of dyspnea, of chest X-ray performed, and of radiological signs of pneumonia (model 1, Table 2). In the second model, in which X-ray related information was not considered, the absence of neurocognitive chronic disorders, the absence of dyspnea, systolic blood pressure, hemoglobin or C-reactive protein levels were independently associated with a delayed diagnosis of pneumonia (model 2, Supplementary Table 4; Supplementary Figure 1).

**Table 2:** Multivariable logistic regression factors associated with a delayed diagnosis of pneumonia after admission to the emergency department (Model 1*).

Variables	Odds Ratio	95% CI	*p*-value
Sex (male vs. female)	1.837	0.963–3.508	0.065
Chronic neurocognitive disorders (yes vs. no)	0.343	0.139–0.848	0.021
Dyspnea (yes vs. no)	0.359	0.187–0.686	0.002
Heart rate (/min)	0.989	0.975–1.003	0.112
C-reactive protein (mg/L)	0.998	0.995–1.003	0.252
Hemoglobin (g/dl)	0.900	0.780–1.039	0.152
Chest X-ray with signs of pneumonia according to the emergency physician’s medical report (yes vs. no)	0.016	0.005–0.048	<0.001
Chest X-ray performed (yes vs. no)	0.415	0.209–0.824	0.012

NB: * Model 1 including chest X-ray as a potential confounding variable

### Outcomes of patients according to the early diagnosis of pneumonia in the ED

Patients with a delayed diagnosis required oxygen less frequently (54 vs. 77%; *p* < 0.001) and were less frequently treated with antibiotics in the ED (34 vs. 75%; *p* < 0.001). In-hospital mortality was significantly lower for patients with a delayed diagnosis than in patients diagnosed earlier (5.4 vs. 13.9%; *p* = 0.045; Table 3). However, after excluding patients with treatment limitations, and when adjusting on confounding variables including initial severity, a delayed diagnosis of pneumonia was not associated with in-hospital mortality (*p* = 0.234) and was not retained in the final prognostic model. Conversely, hemoglobin count, age, Glasgow score, body temperature > 38°C and oxygen flow rate were independently associated with in-hospital mortality (Supplementary Table 5).

**Table 3:** Therapeutics received and outcomes of patients according to the early diagnosis of pneumonia at admission to the emergency department.

	All	Early	Delayed	*p*-value
	*n* = 435	*n* = 361	*n* = 74	
Therapeutics received in ED				
Oxygen administration, *n* (%)	318 (73.0)	278 (77.0)	40 (54.0)	<0.001
Oxygen requirement				<0.001
No oxygen	117 (37)	83 (23)	34 (46)	
1–3 L/min	131 (30)	110 (30)	21 (28)	
4–10 L/min	119 (27)	104 (29)	15 (20)	
>10 L/min or (non)invasive ventilation	68 (16)	64 (18)	4 (5)	
Non-invasive ventilation, *n* (%)	34 (7.8)	27 (7.5)	7 (9.5)	0.563
Invasive ventilation, *n* (%)	3 (0.7)	1 (0.3)	2 (2.7)	0.022
Vasopressors, *n* (%)	2 (0.5)	0	2 (2.7)	0.002
Antibiotics, *n* (%)	294 (68)	269 (75)	25 (34)	<0.001
Treatment limitation, *n* (%)	38 (9.7)	37 (11.4)	1 (1.5)	0.014
Outcomes				
Intensive care unit admission, *n* (%)	121 (27.8)	102 (28.3)	19 (25.7)	0.652
Hospitalization-free days during the 30 first days, median (IQR)	22 (14-26)	22 (13-26)	21 (16-25)	0.300
Antibiotics-free days during the 30 first days, median (IQR)	22 (19-24)	22 (18-23)	22 (20-24)	0.108
In-hospital mortality, *n* (%)	54 (12.4)	50 (13.9)	4 (5.4)	0.045

## Discussion

Our study involving 435 inpatients with a final diagnosis of CAP after admission to the ED yielded 4 main results. First, 17% of patients had a delayed diagnosis of pneumonia after ED admission. Second, patients with a delayed diagnosis had a less symptomatic and severe clinical presentation and less frequently had radiological signs of pneumonia on chest X-ray. Third, patients with a delayed diagnosis less frequently received antibiotics in the ED. Finally, after adjusting on initial severity, a delayed diagnosis of pneumonia was not associated with in-hospital mortality.

Several studies have attempted to analyze diagnostic discrepancies for pneumonia between the ED and other hospital wards (14–16, 20). Among them, two reported overdiagnosis of pneumonia in 27 to 29% of patients in the ED (14, 15), but no study so far has assessed the underdiagnosis of pneumonia in the ED. Here, after excluding hospital-acquired pneumonia, we found that 17% of CAP cases were underdiagnosed after ED care, and sought factors associated with this discrepancy.

Diagnosing CAP currently relies on the combination of systemic and lower respiratory tract signs of infection associated with new infiltrates on chest imaging (21). The diagnosis remains challenging since clinical presentation can be incomplete or atypical. For instance, we observed that patients with a delayed diagnosis had a less symptomatic presentation (less fever and dyspnea) and a less severe disease (lower oxygen flow rate requirement and marginally lower quick-SOFA score). Classically, caution should be exercised when diagnosing CAP in older patients and those who are immunocompromised because they may not exhibit typical symptoms such as fever (22). However, we did not find that patients with a delayed diagnosis were older or more frequently considered as immunocompromised, but they had significantly more chronic liver diseases and fewer chronic neurocognitive disorders. We acknowledge that the diagnosis of CAP in patients suffering from chronic liver diseases (and even more when complicated with cirrhosis) can be difficult since some patients can present altered consciousness and an alternative diagnosis (e.g., ascitic fluid infection). However, clinicians might be more considerate of patients suffering from neurocognitive disorders for whom pneumonia (including aspiration pneumonia) is a frequent complication. Previous work has reported that 11% of patients with cognitive impairment were hospitalized at least once with pneumonia over a period of 10 years (23). Finally, among the main reasons why patients presented to the ED, a fall was significantly more frequently reported among patients with a delayed diagnosis. We could assume that pulmonary contusion may have gone unrecognized in ED, with a delayed development of pneumonia.

Then, it can be assumed that fast track ED before admission of some patients to the ward may explain that the diagnosis of pneumonia was made in the downstream ward. However, we observed the opposite, namely that patients with a delayed diagnosis of pneumonia stayed longer in the ED, despite a lesser severity. it is likely that the diagnostic uncertainty might have contributed to longer emergency room stays. However, other confounding factors may have contributed to this difference.

In our study, the absence of obvious radiological signs of pneumonia was independently associated with a delayed diagnosis of CAP. Chest X-ray is a key examination for establishing lung involvement, but it lacks sensitivity and specificity and can be misinterpreted (24). In the prospective ESCAPED study, CT-scan revealed a lung infiltrate in one third of patients admitted to the ED with clinically suspected CAP and without infiltrates on chest X-ray, and was associated with earlier initiation of antibiotics (25). The authors suggested that early CT scan in the ED could improve CAP diagnosis and clinical management, but these benefits should be balanced by the radiations induced by the scan and the additional costs. Taken together, these data suggest the need to improve the radiological diagnosis of pneumonia in EDs. One solution could be to use low-dose CT, which could be effective in reducing over- or underdiagnosis of pneumonia in the ED but while maintaining low radiation levels (25, 26). In addition, lung ultrasound is increasingly used as a diagnostic tool for pneumonia, providing a sensitivity and specificity as high as 92 and 93%, respectively, according to a meta-analysis of studies conducted in EDs (27). However, no randomized trial has compared the performance of chest X-ray, CT scan and lung ultrasound in patients with suspected pneumonia (28).

It has been shown that diagnostic errors in the ED are associated with increased in-hospital mortality (6). In addition, a prospective multicenter study involving 37 EDs found that, among patients with infection, a misidentification of the infection site was associated with a >10% increase in in-hospital mortality (7). In our study, one third of patients with a delayed diagnosis of pneumonia received antibiotics as early as the ED. The benefit of early antibiotic administration has been previously demonstrated in CAP patients (4). However, this must be counterbalanced with the need to limit antibiotic initiation.

Two thirds of patients with a delayed diagnosis did not receive early antibiotic therapy in our study. Despite this delay in antibiotic administration, we did not observe a worse outcome in this latter group of patients. The delayed diagnosis might reflect less severe forms, rather than masked signs and/or underlying immunodepression, especially since immunocompromised patients were infrequent in our study and equally distributed in the 2 groups. On the other hand, it might reflect earlier management in the natural evolution of the condition. As a consequence, a delayed diagnosis of CAP was associated with a delay in antibiotic initiation, but without negative consequences on clinical outcomes. Accordingly, our data could help reinforce the idea that, in the absence of severity, antibiotics may be safely delayed when a diagnosis of CAP is not established in the ED, even though CAP is not totally ruled out.

Our study has several limitations. First, in addition to its retrospective nature, outpatients discharged from the ED were not included though they may contribute to a more accurate representation of diagnosis of CAP in the ED. While the diagnosis cannot be guaranteed in the absence of thoracic CT scan in all patients, medical files from both the admission to the ED and the subsequent hospitalization were reviewed by a trained clinician. In addition, this is a real-life study in which we considered the diagnosis made by the clinician according to clinical and radiological signs of pneumonia. Finally, this study was conducted prior to the COVID-19 pandemic, so the results cannot be generalized to SARS-CoV-2 pneumonia.

## Conclusion

Our study showed that most individuals who are hospitalized with CAP are diagnosed in the ED. Delayed diagnosis after ED care is thus uncommon and associated with less severe clinical presentation and no obvious signs of pneumonia on chest X-ray. This may reflect that the patient is presenting early-stage disease and/or a lower severity rather than masked signs and/or underlying immunodepression. A delayed diagnosis of CAP was associated with a delayed antibiotic initiation, but without negatively influencing clinical outcome. In line with antibiotic stewardship, these findings strengthen the hypothesis that antibiotics may be safely delayed when the diagnosis of CAP is not established in the ED, even if the condition is not ruled out.

## Data availability statement

The original contributions presented in the study are included in the article/Supplementary material, further inquiries can be directed to the corresponding author.

## Ethics statement

Study protocol and data collection were registered with the CNIL (*Commission nationale de l’informatique et des liberté*s) and are in accordance with French (Loi Informatique et Liberté n°78-17 du 6 janvier 1978) and European (GRPD EU 2016/679) regulations on data protection and patient information (Commitment of compliance MR004 n°2210228 of 3 December 2018).

## Author contributions

MBo and MBl concept, design, and drafting of the manuscript. MBo, MV, and MBl recruitment of patients. MBo, CB, MV, and MBl acquisition, analysis, or interpretation of data. MBo, CB, FM, TS, MV, LP, PR, and MBl critical revision. CB and MBl supervision. All authors contributed to the article and approved the submitted version.

## Conflict of interest

The authors declare that the research was conducted in the absence of any commercial or financial relationships that could be construed as a potential conflict of interest.

## Publisher’s note

All claims expressed in this article are solely those of the authors and do not necessarily represent those of their affiliated organizations, or those of the publisher, the editors and the reviewers. Any product that may be evaluated in this article, or claim that may be made by its manufacturer, is not guaranteed or endorsed by the publisher.

## Abbreviations

CAP: Community-acquired pneumonia
COPD: *Chronic obstructive pulmonary disease*
COVID-19: Coronavirus disease 2019
ED: Emergency department
IQR: Interquartile range
PMSI: *Programme de médicalisation des systèmes d’information*
SD: Standard deviation
SOFA: Sepsis-related Organ Failure Assessment
