# Supplementary material for: Delayed diagnosis of pneumonia in the emergency department: factors associated and prognosis
Source: Front Med (Lausanne). 2023 May 12;10:1042704. doi: 10.3389/fmed.2023.1042704 (PMC10213245; doi:10.3389/fmed.2023.1042704)
Supplement: Supplementary file 1 [file Data_Sheet_1.docx]

**Delayed diagnosis of pneumonia in the emergency department: factors associated and prognosis.**

**-**

**Supplemental data**

**Supplemental Table 1.** Main reasons why patients presented to the emergency department.

|  |  | **All** | **Early** | **Delayed** | ***p-value*** |
| --- | --- | --- | --- | --- | --- |
|  |  | **n=435** | **n=361** | **n=74** |  |
| Dyspnea, n (%) |  | 203 (47) | 173 (48) | 30 (40) | 0.246 |
| Fever, n (%) |  | 90 (21) | 73 (20) | 17 (23) | 0.595 |
| Altered general condition, n (%) |  | 44 (10) | 33 (9) | 11 (15) | 0.137 |
| Fall, n (%) |  | 42 (10) | 30 (8) | 12 (16) | 0.036 |
| Chest pain, n (%) |  | 38 (9) | 35 (10) | 3 (4) | 0.117 |
| Cough, n (%) |  | 36 (8) | 33 (9) | 3 (4) | 0.148 |
| Desaturation, n (%) |  | 32 (7) | 28 (8) | 4 (5) | 0.480 |
| Impaired consciousness, confusion, n (%) |  | 22 (5) | 18 (5) | 4 (5) | 1.000 |
| Digestive disorders, n (%) |  | 18 (4) | 14 (4) | 4 (5) | 0.779 |
| Pneumonia, n (%) |  | 13 (3) | 13 (4) | 0 | 0.200 |
| Malaise, n (%) |  | 10 (2) | 8 (2) | 2 (3) | 1.000 |
| Abdominal pain, n (%) |  | 7 (2) | 7 (2) | 0 | 0.484 |
| Stroke, n (%) |  | 6 (1) | 4 (1) | 2 (3) | 0.600 |
| Hypotension, n (%) |  | 4 (1) | 3 (1) | 1 (1) | 1.000 |
| Hemoptysis, n (%) |  | 3 (1) | 2 (1) | 1 (1) | 1.000 |
| Epilepsy, n (%) |  | 3 (1) | 3 (1) | 0 | 0.987 |
| Headaches, n (%) |  | 2 (0.5) | 2 (1) | 0 | 1.000 |
| Cardiac failure, n (%) |  | 2 (0.5) | 2 (1) | 0 | 1.000 |
| Other reasons, n (%) |  | 14 (3) | 7 (2) | 7 (9) | 0.003 |

**NB:** other reasons were: weakness of the lower limbs, bacteremia, metabolic disorders, dizziness, suspected pulmonary embolism, troponin increase, gastric tube removal (n=2), bone fracture, tachycardia, cervical pain, and suspected CO intoxication.

**Supplemental Table 2.** Alternative diagnoses made in the **e**mergency department for the 74 patients hospitalized with a discharge diagnosis of community-acquired pneumonia (delayed diagnosis).

|  | **N = 74** |
| --- | --- |
| **Respiratory-related diagnosis** |  |
| Bronchitis/exacerbation of COPD or asthma | 13 |
| Viral respiratory infection with no pneumonia | 10 |
| Dyspnea with no etiological diagnosis | 4 |
| Thoracic pain with no etiological diagnosis | 2 |
| Other lung disorder (neoplasia, atelectasis) | 2 |
| **Extra-respiratory-related diagnosis** |  |
| Unexplained fever | 13 |
| Fall with traumatic consequences | 7 |
| Heart failure | 6 |
| Kidney or metabolic dysfunction | 5 |
| Digestive disorder | 3 |
| Extra-respiratory infection (urinary, skin) | 3 |
| Neurological disorder | 2 |
| Miscellaneous | 4 |

**Supplemental Table 3.** Microbiological findings of patients

|  | **All** | **Early** | **Delayed** | ***P-value*** |
| --- | --- | --- | --- | --- |
|  | **n=435** | **n=361** | **n=74** |  |
| Blood cultures, n (%) | 358 (82.3) | 298 (82.5) | 60 (81.1) | 0.763 |
| Positive blood cultures, n (%) | 17 (3.9) | 14 (3.9) | 3 (4.1) | 0.947 |
| Respiratory sample, n (%) | 65 (14.9) | 51 (14.1) | 14 (18.9) | 0.292 |
| Positive respiratory sample, n (%) | 36 (55.4) | 26 (51.0) | 10 (71.4) | 0.173 |
| *Legionella* Sp1 antigenuria, n (%) | 163 (37.5) | 149 (41.3) | 14 (18.9) | <0.001 |
| Positive *Legionella* Sp1 antigenuria, n (%) | 6 (3.7) | 6 (4.0) | 0 | 0.444 |
| Respiratory virus testing with PCR, n (%) | 276 (63.4) | 229 (63.4) | 47 (63.5) | 0.990 |
| Positive respiratory virus testing with PCR, n (%) | 80 (29.0) | 65 (28.5) | 15 (31.3) | 0.704 |

NB: PCR : polymerase chain reaction; Sp1: Serogroup pneumophila 1

**Supplemental Table 4.** Multivariable logistic regression factors associated with a delayed diagnosis of pneumonia after admission to the emergency department (Model 2*).

| **Variables** | **Odds Ratio** | **95% CI** | **p-value** |
| --- | --- | --- | --- |
| Sex (male vs. female) | 1.428 | 0.816 - 2.500 | 0.212 |
| Chronic liver disease (yes vs no) | 2.617 | 0.859 - 7.974 | 0.091 |
| Neurocognitive disorders (yes vs no) | 0.416 | 0.189 - 0.913 | 0.029 |
| Inflammatory/immune disorders (yes vs no) | 1.050 | 0.318 - 3.468 | 0.936 |
| Temperature ≥ 38°C (yes vs no) | 0.854 | 0.488 - 1.494 | 0.580 |
| Dyspnea (yes vs no) | 0.466 | 0.271 - 0.800 | 0.006 |
| Systolic blood pressure (mmHg) | 1.013 | 1.002 - 1.025 | 0.017 |
| Heart rate (/min) | 0.995 | 0.982 - 1.008 | 0.457 |
| C-reactive protein (mg/L) | see Suppl. Figure 2 | | 0.013 |
| Hemoglobin (g/dL) | 0.878 | 0.771 - 0.999 | 0.048 |
| Neutrophil count (x10^3^/mm^3^) | 1.022 | 0.970 - 1.076 | 0.416 |

NB: * Model 2 not including chest X-ray as a potential confounding variable

**Supplemental Table 5.** Multivariable logistic regression factors associated with in-hospital mortality among 397 inpatients with CAP and no treatment limitation.

| **Variables** | **Odds Ratio** | **95% CI** | **p-value** |
| --- | --- | --- | --- |
| Age (years) | 1.090 | 1.048 - 1.134 | <0.001 |
| Cancer (yes/no) | 6.641 | 2.656 - 16.604 | <0.001 |
| Temperature (zenith) ≥ 38°C (yes/no) | 0.333 | 0.152 - 0.732 | 0.006 |
| Oxygen requirement, class (1-4) | 3.596 | 1.664 - 7.768 | 0.001 |
| Glasgow score | 2.404 | 1.078 - 5.362 | 0.032 |
| Hemoglobin (g/dl), transformation 1 | 0.078 | 0.020 - 0.304 | <0.001 |
| Hemoglobin (g/dl), transformation 2 | 43.397 | 6.191 - 304.200 | <0.001 |

NB: Oxygen requirement class (1: no oxygen; 2: 1-3 L/min; 3: 4-10 L/min; 4: >10 L/min or (non)invasive ventilation.

**Supplemental Figure 1.** Polynomial representation of C-reactive protein concentrations according to the diagnosis of community acquired pneumonia in the ED (early or delayed).

**Note:** we observed a stable effect until a C-reactive protein (CRP) of 400 mg/L with a decrease in the probability of having a delayed diagnosis for each one-unit increase in CRP, this effect being cancelled for higher concentrations of CRP (possible outliers).
